# Painful stimulation increases functional connectivity between supplementary motor area and thalamus in patients with small fibre neuropathy

**DOI:** 10.1002/ejp.4720

**Published:** 2024-08-28

**Authors:** Sebastian Scheliga, Maike F. Dohrn, Thilo Kellermann, Angelika Lampert, Roman Rolke, Barbara Namer, Greta Z. Peschke, Nortje van den Braak, Annette Lischka, Marc Spehr, Han‐Gue Jo, Ute Habel

**Affiliations:** ^1^ Department of Psychiatry, Psychotherapy and Psychosomatics, Medical Faculty RWTH Aachen University Aachen Germany; ^2^ Scientific Center for Neuropathic Pain Aachen, SCNAachen Uniklinik RWTH Aachen University Aachen Germany; ^3^ Department of Neurology, Medical Faculty RWTH Aachen University Aachen Germany; ^4^ Institute of Neuroscience and Medicine: JARA‐Institute Brain Structure Function Relationship (INM 10) Research Center Jülich, Wilhelm‐Johnen‐Straβe Jülich Germany; ^5^ Institute of Neurophysiology, Medical Faculty RWTH Aachen University Aachen Germany; ^6^ Department of Palliative Medicine, Medical Faculty RWTH Aachen University Aachen Germany; ^7^ Research Group Neuroscience, Interdisciplinary Centre for Clinical Research, Medical Faculty RWTH Aachen University Aachen Germany; ^8^ Institute for Human Genetics and Genomic Medicine, Medical Faculty RWTH Aachen University Aachen Germany; ^9^ Department of Chemosensation RWTH Aachen University, Institute for Biology II Aachen Germany; ^10^ School of Computer Science & Engineering Kunsan National University Gunsan Republic of Korea

## Abstract

**Background:**

The lead symptom of small fibre neuropathy (SFN) is neuropathic pain. Recent functional magnetic resonance imaging (fMRI) studies have indicated central changes in SFN patients of different etiologies. However, less is known about brain functional connectivity during acute pain processing in idiopathic SFN.

**Methods:**

We conducted fMRI with thermal heat pain application (left volar forearm) in 32 idiopathic SFN patients and 31 healthy controls. We performed functional connectivity analyses with right supplementary motor area (SMA), left insula, and left caudate nucleus (CN) as seed regions, respectively. Since pathogenic *gain‐of‐function* variants in voltage gated sodium channels (Nav) have been linked to SFN pathophysiology, explorative connectivity analyses were performed in a homogenous subsample of patients carrying rare heterozygous missense variants.

**Results:**

For right SMA, we found significantly higher connectivity with the right thalamus in SFN patients compared to controls. This connectivity correlated significantly with intraepidermal nerve fibre density, suggesting a link between peripheral and central pain processing. We found significantly reduced connections between right SMA and right middle frontal gyrus in patients with Nav variants. Likewise, connectivity between left CN and right frontal pole was decreased.

**Conclusions:**

Aberrant functional connectivity in SFN is in line with previous research on other chronic pain syndromes. Functional connectivity changes may be linked to SFN, highlighting the need to determine if they result from peripheral changes causing abnormal somatosensory processing. This understanding may be crucial for assessing their impact on painful symptoms and therapy response.

**Significance statement:**

We found increased functional connectivity between SMA and thalamus during painful stimulation in patients with idiopathic SFN. Connectivity correlated significantly with intraepidermal nerve fibre density, suggesting a link between peripheral and central pain processing. Our findings emphasize the importance of investigating functional connectivity changes as a potential feature of SFN.

## 1 INTRODUCTION

Small fibre neuropathy (SFN) is a multifaceted neurological condition primarily affecting small sensory, thinly or unmyelinated nerve fibres. SFN is characterized by neuropathic pain and autonomic symptoms caused by dysfunction and degeneration of thinly myelinated Aδ‐fibres and unmyelinated C‐fibres (Devigili et al., 2008; Sopacua et al., 2019). Consequently, the evaluation of intraepidermal nerve fibre density (IENFD) and quantitative sensory testing (QST) play a pivotal role in confirming the diagnosis (Devigili et al., 2008; Hoitsma et al., 2004, 2003; Lauria et al., 2010b; Lauria, Bakkers, et al., 2010). Traditionally, SFN has been discussed as a condition primarily affecting the peripheral nervous system, with only a limited number of MRI studies exploring its potential impact on the brain (e.g. Chao et al., 2021; Hsieh et al., 2015; Tseng et al., 2015).

Previous functional magnetic resonance imaging (fMRI) studies found activation of pain‐related brain regions during heat pain (Hsieh et al., 2015). Analyses revealed functional connectivity changes in patients with SFN that was correlated with the amount of nerve fibre reduction (e.g., Hsieh et al., 2015; Tseng et al., 2015). These studies point to a link between peripheral nerve fibres and brain functional changes in SFN patients. However, to date, it remains unclear whether corresponding effects can be reproduced in an idiopathic SFN patient population.

Recent discoveries have unveiled *gain‐of‐function* variants in genes encoding voltage‐gated sodium channel (Nav) subunits, particularly Nav1.7, Nav1.8, and Nav1.9 (gene names: *SCN9A*, *SCN10A*, and *SCN11A*), among SFN patients (Bennett et al., 2019; Faber et al., 2012a; Faber et al., 2012b). *Gain‐of‐function* mutations may play a role in peripheral neuropathy (Faber et al., 2012b). However, our understanding of central pain markers in SFN patients with Nav gene variants remains limited. Therefore, our objective is to investigate brain activity and functional connectivity in SFN patients with and without Nav variants and healthy subjects.

The aim of our study was to investigate altered functional connectivity in pain‐processing brain structures during thermal heat pain application in patients with idiopathic SFN. Further, we aimed to assess the clinical significance of these functional changes by analysing correlations between neural findings and clinical parameters, with a specific focus on IENFD. According to previous studies, we expected that connectivity strength may be linked to IENFD in particular.

Furthermore, we conducted explorative analyses on brain activity and functional connectivity on a subsample of patients with heterozygous missense variants in *SCN9A*, *SCN10A*, and *SCN11A*, the pathogenicity of which remains undetermined at present.

## 2 METHODS

### 2.1 Participants

We examined a total of 67 participants, including 35 patients with SFN and 32 healthy individuals serving as controls (HC). However, four subjects (one HC and three patients) had to be excluded from further analyses, due to extensive head motion during the scanning session. The final study sample consisted of 32 SFN patients and 31 HC. From these subjects, 26 SFN patients and 25 HC have already been considered for the sample in our previous VBM study (Scheliga et al., 2024). All participants were evaluated at the University Hospital Aachen, RWTH Aachen University, Germany, and did provide written consent before enrolment. The study was approved from the local institutional review board (EK215‐19) and conducted in accordance with the principles of the Declaration of Helsinki.

All patients underwent examinations conducted by a team of trained physicians. SFN diagnosis was based on criteria published by Devigili and colleagues in 2008 (Devigili et al., 2008). All patients had typical SFN symptoms, including neuropathic pain and additional sensory and/or autonomic symptoms, and additional diagnostic signs of SFN, which was either a reduction of intraepidermal nerve fibre densities by skin biopsy (Lauria et al., 2010) and/or signs of A‐delta‐/C‐nerve fibre dysfunction by QST (Dohrn et al., 2022; Rolke et al., 2006a; Rolke et al., 2006b). Large fibre polyneuropathy was ruled out by nerve conduction studies of the tibial and sural nerves prior to study inclusion. Detailed neurological assessments (Dohrn et al., 2022) were conducted on all patients to document the presence or absence of spontaneous pain (such as thermal hyperalgesia and mechanical allodynia), as well as any paresthesia or dysesthesia reported by the patients. Nerve conduction studies were performed to rule out large fibre neuropathy. Patients with any clinical evidence of brain or spinal cord disease were excluded from the study to ensure that the observed symptoms were solely related to SFN and not due to central nervous system involvement from other systemic diseases (Chao et al., 2021; Hsieh et al., 2015). Similarly, patients with secondary SFN resulting from conditions such as diabetes mellitus, alcoholism, vitamin B12 deficiency, or chemotherapy were excluded from the study. Information was gathered regarding the onset of symptoms, the duration of illness, and the patient's subjective experience of pain. This included the administration of the painDETECT questionnaire (Freynhagen et al., 2006; Freynhagen et al., 2016) and the Numeric Rating Scale (NRS) (Hartrick et al., 2003; Krebs et al., 2007; Williamson & Hoggart, 2005) for pain. Demographic and clinical data for all participants can be found in Table 1. The participants were categorized as male or female based on a binary sex definition (assigned at birth). In terms of racial background, the control group consisted exclusively of white (Caucasian) individuals while the patient cohort included one black individual and 31 Caucasian individuals. Additional details regarding the patients' medication status are provided in the supplementary material (Table S1).

**TABLE 1:** Participant demographic and clinical data.

	Healthy controls	SFN patients	*p*‐value
N (m/f)	31 (16/15)	32 (14/18)	0.617
Age^a^	42.32 (16.93)	43.38 (11.01)	0.385
Symptom duration (years)^a^	—	7.81 (5.04)	—
IENFD^a^	—	4.35 (2.63)	—
BMI^a^	—	24.99 (4.12)	—
CDT^a^, ^b^ (test)	0.42 (0.27)	0.58 (0.25)	0.023
CDT^a^, ^b^ (control)	0.08 (0.25)	0.49 (0.31)	<0.001
WDT^a^, ^b^ (test)	0.62 (0.31)	0.83 (0.24)	0.006
WDT^a^, ^b^ (control)	0.29 (0.25)	0.47 (0.26)	0.010
CPT ^a^ (test)	11.67 (9.82)	11.40 (7.90)	0.909
CPT ^a^ (control)	12.16 (9.21)	12.68 (8.33)	0.822
HPT ^a^ (test)	45.88 (2.38)	44.69 (4.08)	0.181
HPT ^a^ (control)	45.29 (3.07)	42.28 (6.17)	0.022
painDETECT^a^	—	11.97 (8.21)	—
NRS^a^	—	3.55 (2.16)	—

*Note*: The values for the healthy subjects were taken from a sex‐, age‐, and region‐matched reference sample.

Abbreviations: BMI, body mass index; CDT, cold detection threshold; CPT, cold‐pain threshold; f, female; HPT, heat‐pain threshold; IENFD, intraepidermal nerve fibre density; m, male; NRS, numeric rating scale; WDT, warm detection threshold.

^a^ Mean (Standard deviation).

^b^ Logarithmically transformed QST‐thresholds are shown for test‐ and control‐area, respectively.

Methods and results of genetic testing in the patients have been described previously (Scheliga et al., 2024). In summary, whole exome sequencing identified nine subjects with a total of ten rare variants in the SFN‐related genes *SCN9A*, *SCN10A*, or *SCN11A*. In four patients, a variant in the *SCN11A* gene (which encodes Nav1.9) was found, three patients carried a variant in the *SCN10A* gene (Nav1.8), and three patients had a variant in the *SCN9A* gene (Nav1.7). Notably, one subject carried a variant in each the *SCN9A* and *SCN10A* genes. Thus, the sample comprised nine patients with ten genetic variants in total. All identified variants were officially classified as variants of uncertain significance based on the ACMG criteria (Richards et al., 2015). Further details and descriptions of these genetic variants can be found in the supplementary material (Table S2).

### 2.2 Stimuli and task

#### 2.2.1 Thermal threshold detection

Thermal stimuli were administered using the Pain & Sensory Evaluation System (PATHWAY model, Medoc, Israel) which is compatible with the MR environment. The system allows for the delivery of temperatures ranging from 0 to 55°C with a heating rate of 70°C/sec and cooling rate of 40°C/sec. The thermode was placed on the participants' left forearm.

Prior to the fMRI task, the PATHWAY thermotester was used to determine the individual thermal thresholds for warm and hot painful stimuli for each participant by placing a thermode (PATHWAY's movable skin contact tool) to the volar forearm. This procedure was adapted from the QST manual following the established protocol of the German Research Council on Neuropathic Pain, which has been introduced in 2006 and has now become the European standard for studies and trials on neuropathic pain (Rolke et al., 2006a; Rolke et al., 2006b). Further details on QST can be found in section 2.8 ‘QST measures in patients with SFN’. The warm detection threshold (WDT) represents the temperature at which the participant first sensed a warming sensation on the left forearm. The heat pain threshold (HPT) represents the temperature at which the participant indicated that the temperature became painful. To calibrate these thresholds, the thermode was gradually heated until the participant detected either a warm or painful stimulus. This procedure was repeated four times for each threshold. Mean temperature values at which the thresholds were reached were defined as warm threshold and heat threshold, respectively. To avoid excessive heat exposure that could potentially lead to burns or tissue damage, the maximum temperature applied was set at 48°C. It should be noted that although a complete QST was conducted for each patient on a different day, we detected WDT and HPT once more immediately prior to the MRI session. This is because thermal thresholds may also be influenced by daily factors and varying psychological states (Starr et al., 2010). To avoid confusion, from here on, thresholds detected immediately before the fMRI session will be referred to as warm threshold and heat threshold, while thresholds detected during QST (data that has been collected on a separate day) will be referred to as WDT and HPT.

#### 2.2.2 fMRI task with thermal stimulation

**FIGURE 1:**
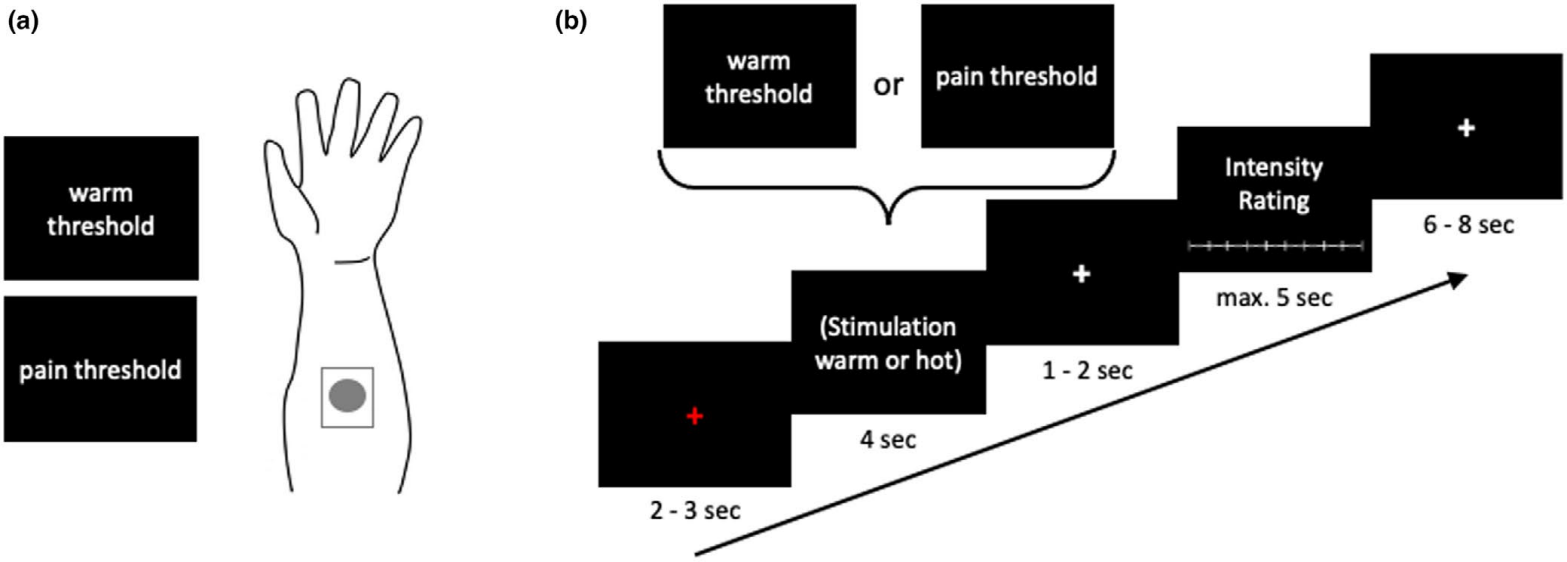
Thermal threshold detection and fMRI task. (a) Thermode was placed on the participants' left forearm to detect individual thresholds of warmth and painful heat for each participant. (b) During the scanning session, participants experienced warm and heat stimuli in a randomized order and rated their intensity using an 11‐point Likert scale. Each trial included an alert cue, stimulus delivery, and rating phase, with a brief pause between trials. The session comprised 40 trials and lasted approximately 13 min.

During the scanning session, warm and hot stimuli were administered in a randomized order. After each stimulus, participants used the MR‐compatible LUMItouch response system (LumiTouch, Photon Control, Burnaby, Canada), which was placed under their right hand, to rate the intensity of the stimulus on an eleven‐point Likert scale ranging from 0 to 10, 0 indicating “not intense at all” and 10 indicating “a very intense sensation”. Each trial consisted of an alert cue displayed for two to three seconds, followed by the delivery of the stimulus for four seconds, and finally by the participants' rating of the stimulus within another few seconds. A pause of six to eight seconds separated each trial from the next one (Figure 1). In total, 40 trials were conducted during the session, that added up to a total of approximately 13 min. A functional MRI was recorded throughout the entire paradigm.

### 2.3 Data acquisition

MRI was conducted using a 3 Tesla MR scanner (MAGNETOM Prisma, Siemens Medical Systems, Germany). For structural imaging, magnetization‐prepared rapid acquisition gradient echo (MPRAGE) was utilized. MPRAGE scan parameters were as follows: repetition time (TR) of 2000 ms, echo time (TE) of 3.03 ms, voxel size 1.0 × 1.0 × 1.0 mm^3^, flip angle of 9°, 176 slices, and matrix size of 256 × 256. MPRAGE duration was 4 min and 16 s.

Conducting functional measurements, blood oxygen level‐dependent (BOLD) contrast images were acquired. For BOLD images, the following parameters were defined: TR of 2000 ms, TE of 28 ms, voxel size of 3.0 × 3.0 × 3.0, flip angle of 77°, 34 slices, and matrix size of 64 × 64. Duration of the functional recording was 13 min and 26 s. Also, field maps were obtained using the following parameters: TR of 526 ms, TE of 4870 ms, voxel size of 3.0 × 3.0 × 3.0 mm^3^, flip angle of 60°, 50 slices, and matrix size of 64 × 64. Field map recording took approximately 1 min and 30 s.

### 2.4 Data preprocessing and denoising

Functional and anatomical data was preprocessed using a flexible preprocessing pipeline (Nieto‐Castanon & Whitfield‐Gabrieli, 2021) including realignment with correction of susceptibility distortion interactions, slice timing correction, outlier detection, direct segmentation, MNI‐space normalization, and smoothing. The functional data was realigned using the SPM realign and unwarp procedure (Andersson et al., 2001), whereby all scans were co‐registered to a reference image (the first scan of the first session) using a least squares approach and a six‐parameter (rigid body) transformation (Friston et al., 1995). Subsequently, the data was resampled using b‐spline interpolation to correct for motion and magnetic susceptibility interactions. Temporal misalignment between different slices of the functional data (acquired in ascending order) was corrected following SPM slice‐timing correction procedure (Henson et al., 1999; Sladky et al., 2011), using sinc temporal interpolation to resample each slice of the BOLD timeseries to a common mid‐acquisition time. Potential outlier scans were identified using ART (Whitfield‐Gabrieli et al., 2011) as acquisitions with framewise displacement above 0.9 mm or global BOLD signal changes above 5 standard deviations (Power et al., 2014), and a reference BOLD image was computed for each subject by averaging all scans excluding outliers. Functional and anatomical data was normalized into standard MNI space, segmented into grey matter, white matter, and cerebrospinal fluid (CSF) ‘tissue classes’, and resampled to 2 mm isotropic voxels following a direct normalization procedure (Calhoun et al., 2017) using SPM unified segmentation and normalization algorithm (Ashburner, 2007; Ashburner & Friston, 2005) with the default IXI‐549 tissue probability map template. Finally, functional data was smoothed using spatial convolution with a Gaussian kernel of 8 mm full width half maximum (FWHM).

In addition, functional data was denoised using a standard denoising pipeline (Nieto‐Castanon, 2020, pp. 83–104) including the regression of potential confounding effects characterized by white matter timeseries (5 CompCor noise components), CSF timeseries (5 CompCor noise components), motion parameters and their first order derivatives (12 factors) (Friston et al., 1996), outlier scans (below 41 factors) (Power et al., 2014), session and task effects and their first order derivatives (6 factors), and linear trends (2 factors) within each functional run, followed by bandpass frequency filtering of the BOLD timeseries (Hallquist et al., 2013) between 0.008 Hz and 0.09 Hz. CompCor (Behzadi et al., 2007; Chai et al., 2012). Noise components within white matter and CSF were estimated by computing the average BOLD signal as well as the largest principal components orthogonal to the BOLD average, motion parameters, and outlier scans within each subject's eroded segmentation masks.

### 2.5 General linear model (GLM) analyses

For each subject, parameter estimates were calculated within the GLM. From the fMRI task, experimental conditions ‘warm threshold’ and ‘heat threshold’ were considered as the two regressors of interest ‘Warm’ and ‘Hot’ along with realignment parameters and the intensity rating as parametric modulation which has not been analysed in the present study. Each parameter estimate was convolved with a canonical hemodynamic response function provided by SPM12. The resulting contrast images were subjected to a 2 × 2 ANOVA with two applied temperatures (Hot and Warm) and two groups (HC and SFN patients). The contrast Hot > Warm was first assessed for HC and patients, separately. After that, a group comparison (HC > SFN patients; SFN patients > HC) was calculated for the same contrast to assess potential group differences during pain processing. Also, we performed explorative subgroup analyses. First, the contrast Hot > Warm was assessed for SFN patients with Nav variants and patients without Nav variants, respectively. Further, the following group comparisons were calculated for this contrast: HC > SFN patients with Nav variants, SFN patients with Nav variants > HC, HC > SFN patients without Nav variants, SFN patients without Nav variants > HC, SFN patients with Nav variants > SFN patients without Nav variants, SFN patients without Nav variants > SFN patients with Nav variants. For all contrasts, a cluster defining threshold of *p* < 0.001 uncorrected in combination with an ad‐hoc extent threshold of 20 voxels was applied. Additionally, a false‐discovery‐rate (FDR) corrected threshold of *p* < 0.05 was applied to correct for multiple comparisons.

### 2.6 Seed‐based connectivity analyses

Based on the GLM findings, the right supplementary motor area (SMA) and left insula were chosen as seed regions for connectivity analyses. These two regions were the only two clusters that also survived an FDR correction (*p* < 0.05) at the cluster level in HC and SFN patients, respectively. Moreover, these were the clusters with the highest t‐values in the respective groups. In a previous VBM study, we found grey matter volume differences between SFN patients and HC in left caudate nucleus (CN) (Scheliga et al., 2024). Therefore, left CN was also defined as a seed. Seed‐based connectivity maps (SBC) were estimated characterizing the patterns of functional connectivity with 164 HPC‐ICA networks (Nieto‐Castanon, 2020, pp. 17–25) and Harvard‐Oxford atlas ROIs (Desikan et al., 2006). Functional connectivity strength was represented by Fisher‐transformed bivariate correlation coefficients derived from a weighted general linear model (weighted‐GLM) (Nieto‐Castanon, 2020, pp. 3–16). This was done separately for each pair of seed and target areas, with the aim of modelling the association between their BOLD signal timeseries. Individual scans were weighted by a boxcar signal characterizing each individual task or experimental condition convolved with an SPM canonical hemodynamic response function and rectified.

Group‐level analyses were performed using a GLM (Nieto‐Castanon, 2020, pp. 26–62). For each individual voxel, a separate GLM was estimated, with first‐level connectivity measures at this voxel as dependent variables (one independent sample per subject and one measurement per task or experimental condition, if applicable), and groups or other subject‐level identifiers as independent variables. Voxel‐level hypotheses were evaluated using multivariate parametric statistics with random‐effects across subjects and sample covariance estimation across multiple measurements. Inferences were performed at the level of individual clusters (groups of contiguous voxels). Cluster‐level inferences were based on parametric statistics from Gaussian Random Field theory (Worsley et al., 1996; Nieto‐Castanon, 2020, pp. 63–82). Results were thresholded using a cluster‐forming *p* < 0.001 voxel‐level threshold, and a FDR corrected *p* < 0.05 at the cluster‐level (Chumbley et al., 2010). To assess functional connectivity on the group level, we calculated the same contrasts as mentioned above (see section 2.5 GLM analyses). This approach is called psychophysiological interaction (PPI) analysis. We used this type of analysis to assess the effect of temperature × group interactions on brain functional connectivity. Thereby, PPI refers to connectivity changes between the seed and the target region (physiological), which is modulated by external stimulation (the psychological variable ‘hot vs. warm’).

### 2.7 Correlation analyses

From seed‐based connectivity analyses, we extracted Fisher‐transformed correlation coefficients for those regions that displayed significant functional connectivity differences between HC and SFN patients in the contrast Hot > Warm. As the right SMA was the sole seed for which a notable connection with the target region in the right thalamus was observed (see Results, Section 3.1.4), its Fisher‐transformed correlation coefficients were extracted for subsequent correlational analyses. This coefficient was then correlated with various clinical parameters. These parameters included IENFD, duration of illness, painDETECT score, NRS score, cold detection threshold (CDT), WDT, cold‐pain threshold (CPT), and HPT. These thresholds from QST were taken from the patients' painful test area and non‐painful control area, respectively. However, in some cases, complete values for clinical parameters were not available, resulting in varying sample sizes for correlation analyses. Notably, in HC, for IENFD, duration of illness, painDETECT scores, and NRS scores the data was not available. Therefore, for these clinical parameters, we considered the patients' data only for correlation analysis.

The sample sizes for correlation analyses were as follows: IENFD (*n* = 26); symptom duration (*n* = 31); NRS (*n* = 28); painDETECT (n = 31); CDT, WDT, CPT, HPT (test and control sites) (*n* = 29). In total, we conducted 12 tests correlating the clinical measures with Fisher transformed correlation coefficients from right thalamus. To perform these analyses, we used Pearson's bivariate correlation analysis in SPSS 27. The significance threshold was set at *p* < 0.05. Bonferroni correction was applied to account for multiple testing.

### 2.8 QST measures in patients with SFN

For the correlation analyses, we utilized z‐transformed values of QST for CDT, WDT, CPT and HPT. To calculate these z‐values, we employed the following formula: Z = (mean patients – mean controls) / standard deviation controls. The logarithmically transformed data (for CDT and WDT) or raw QST data (for CPT and HPT) was used in this calculation. The mean and standard deviation of the control from a HC population were used as reference values. To determine differences between SFN patients and healthy reference subjects in terms of CDT, WDT, CPT, and HPT, we conducted unpaired t‐tests. These tests were performed to compare QST values between the two groups. Significance threshold was set at *p* < 0.05. Healthy reference subjects were carefully matched with SFN patients in terms of age, sex, and the affected body region. QST was performed within the following test areas: left foot (*n* = 22), right foot (*n* = 4), face (*n* = 1), left lower leg (n = 1), left hand (n = 1). We used the following body regions as control areas: left hand (*n* = 19), face (*n* = 3), right hand (n = 4), right foot (n = 1), left upper arm (n = 1), belly (n = 1). Notably, the test area was individually chosen, being clinically relevantly affected by SFN symptoms. Ideally, the control area should not be affected. If all available areas (feet, hands, face) are affected, we chose the least affected area as control area.

## 3 RESULTS

### 3.1 Comparison of healthy controls and SFN patients

#### 3.1.1 Thermal thresholds and intensity rating

**FIGURE 2:**
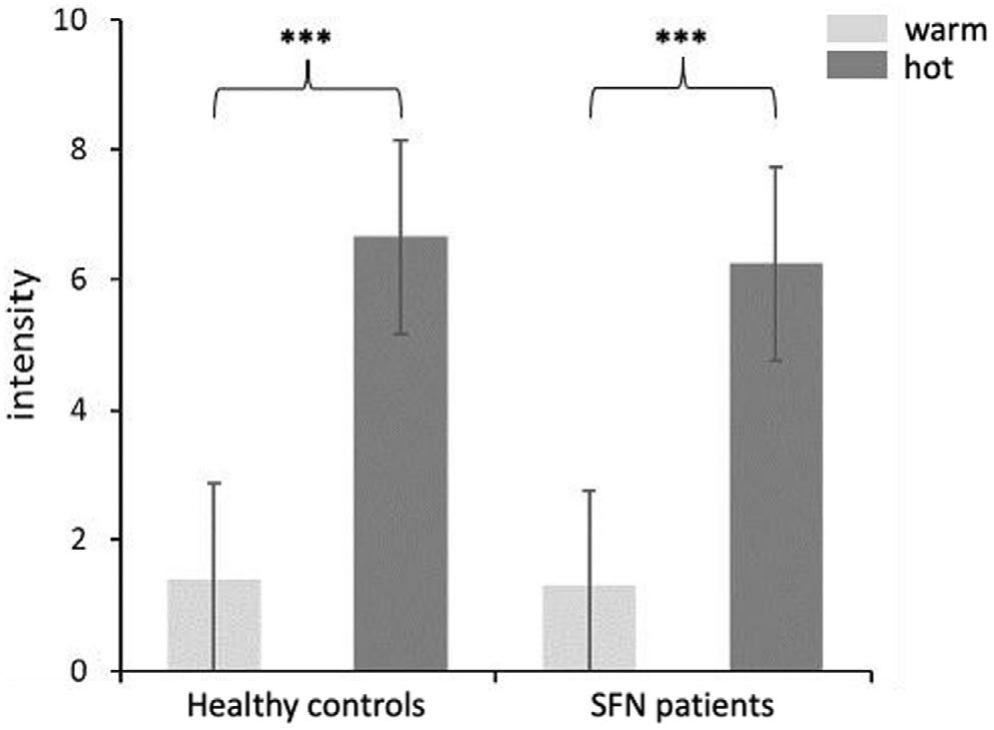
Intensity ratings of thermal stimulation. Displayed are intensity ratings of warm (light grey) and hot painful (dark grey) stimuli in SFN patients and healthy subjects, respectively. Asterisks denote levels of significance: *p < 0.05, ***p < 0.001.

Between HC and SFN patients, we compared the mean warm thresholds and heat thresholds that were detected immediately before the fMRI session. For the warm threshold, we found no significant differences (*p* = 0.122) between HC (*M* = 36.27, *SD* = 1.78) and patients (*M* = 36.88, *SD* = 2.27). Similarly, there were no differences between controls (*M* = 44.31, *SD* = 2.37) and patients (*M* = 44.27, *SD* = 2.96) for the heat threshold (*p* = 0.478). Within each group, the difference between warm threshold and heat threshold was significant (*p* < 0.001, respectively). Notably, in the test area (left volar forearm), some patients reported feelings of numbness (*n* = 4), insensitivity (*n* = 1), or hypersensitivity (*n* = 2). When excluding these patients from the analyses, the respective results did not reveal significant group differences. No significant differences between HC (*M* = 1.41, *SD* = 1.34) and patients (*M* = 1.30, *SD* = 0.98) were found for the intensity rating of warm stimuli (*p* = 0.357). Also, for hot stimuli, intensity rating did not reveal significant differences (*p* = 0.149) between HC (*M* = 6.66, *SD* = 1.53) and patients (*M* = 6.26, *SD* = 1.53). Both, controls and patients showed significantly higher intensity ratings for hot stimuli compared to warm stimuli (*p* < 0.001, respectively) (Figure 2).

#### 3.1.2 GLM

First, we analysed the GLM data (contrast Hot > Warm) for HC subjects and SFN patients, separately. After analysing pain‐related brain activity for each group separately, we compared HC subjects and SFN patients. Note, that none of the analyses survived FDR‐correction for multiple comparisons (*p* < 0.05). Uncorrected GLM results (*p* < 0.001) can be found in the supplement (Table S3). In HC, the contrast Hot > Warm revealed pain‐related activation in right SMA (*p* < 0.001) that survived an FDR‐corrected threshold of *p* < 0.05 at the cluster level. For SFN patients, the analysis yielded pain‐related brain activity in left SFG (*p* = 0.016) and insula (*p* = 0.002) which survived FDR‐correction (*p* < 0.05) at the cluster level.

#### 3.1.3 Seed to voxel connectivity

We conducted seed‐based connectivity analyses for the contrast Hot > Warm (Table 2). Thereby, we defined the following brain structures as seed regions: right SMA, left CN, and insula.

**TABLE 2:** Significant connectivity results (Hot > Warm).

Contrast	Seed	Region	k	Peak MNI coordinates			Peak T score*
				x	y	z	
*HC > SFN patients*	R SMA	R thalamus	90	6	−20	12	−4.40
*HC > SFN patients with Nav variants > HC*	R SMA	R MFG	110	32	28	46	5.60
*HC > SFN patients without Nav variants*	R SMA	L cerebellum (Crus 2)	82	−8	−86	−32	−4.64
*HC > SFN patients with Nav variants*	L CN	R FP	162	20	54	6	5.72
*SFN patients without Nav variants*	L insula	L SPL	70	−40	−40	56	5.16

Abbreviations: CN, caudate nucleus; FP, fontal pole; HC, healty control; L, left; MFG, middle frontal gyrus; R, right; SFN, small‐fibre neuropathy; SMA, supplementary motor area; SPL, superior parietal lobule.

* Results were thresholded at the cluster‐level using a cluster‐forming threshold of *p* < 0.001 uncorrected at the voxel‐level and a familywise corrected p‐FDR <0.05 at the cluster‐level.

**FIGURE 3:**
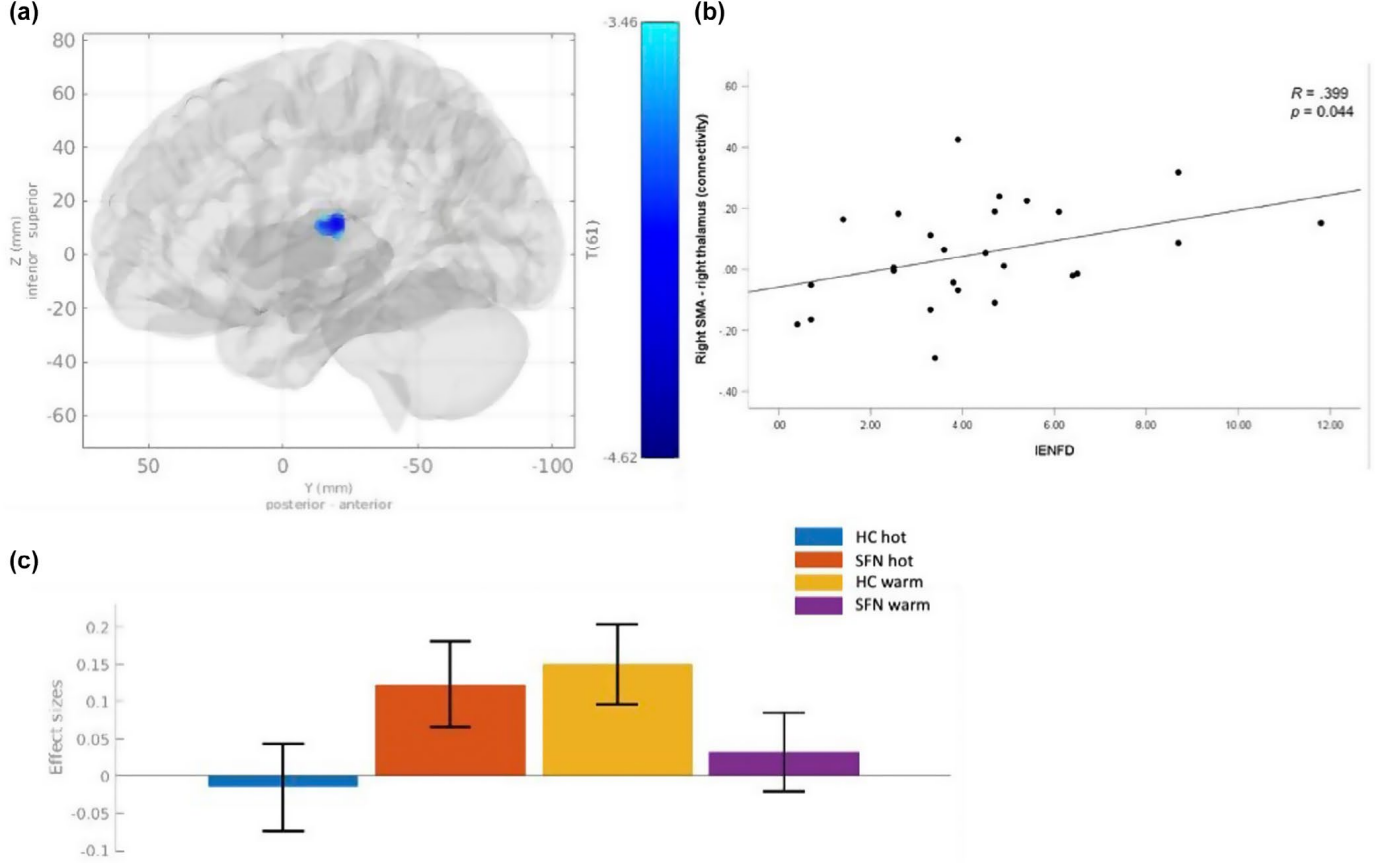
Differences in functional connectivity between SFN patients and HC. (a) Displayed is a brain map with one cluster in right thalamus (blue) showing pain‐related functional connectivity with a predefined right SMA seed region. (b) Bar chart indicates that functional connectivity was increased in SFN patients compared to HC. (c) Scatterplot visualizing a positive correlation between IENFD on the one hand and the estimates for the psychophysiological interaction (PPI) of the SMA‐thalamus connectivity by hot vs. warm stimulation. Correlation was thresholded at *p* < 0.05 (uncorrected). All brain maps were thresholded at voxel level of *p* < 0.001 (uncorrected) and cluster level of *p* < 0.05 (FDR corrected).

Using the right SMA seed, we analysed functional connectivity for HC subjects and for SFN patients, separately. For each group, we did not find significant results. Also, we performed a group comparison (HC > SFN). The analysis revealed significant group differences for one cluster in the right thalamus (Table 2, Figure 3a). The negative t‐value indicates a significantly higher SMA‐thalamus coupling during pain processing in SFN patients compared to HC (Table S3, Figure 3b). In other words, the psychophysiological interaction of the SMA‐thalamus coupling is stronger modulated by the painful stimulation in SFN patients as compared to HC.

### 3.2 Correlation analysis

We conducted correlation analyses, to determine if our connectivity results can be linked to clinical factors in the patient sample. We chose the significant cluster within right thalamus from the Hot > Warm contrast comparing HC and SFN patients (seed region right SMA). We correlated thalamus' Fisher‐transformed correlation coefficient within eight z‐transformed QST subscales (CDT, WDT, CPT, and HPT for both test and control areas, respectively), IENFD, duration of illness, painDETECT score, and NRS score.

None of the correlations survived Bonferroni correction for multiple testing. Both, Bonferroni corrected and uncorrected p‐values can be found in the supplement (Table S4). Notably, uncorrected analysis revealed one significant correlation between SMA‐thalamus connectivity and IENFD (*r* = 0.399, *p* = 0.044) (Figure 3c). As one of our hypotheses, we expected a link between pain related brain activity and reduced IENFD which is a typical clinical feature for the SFN diagnosis. Therefore, we decided to report the uncorrected finding for this correlation only.

### 3.3 QST measures in SFN patients

**FIGURE 4:**
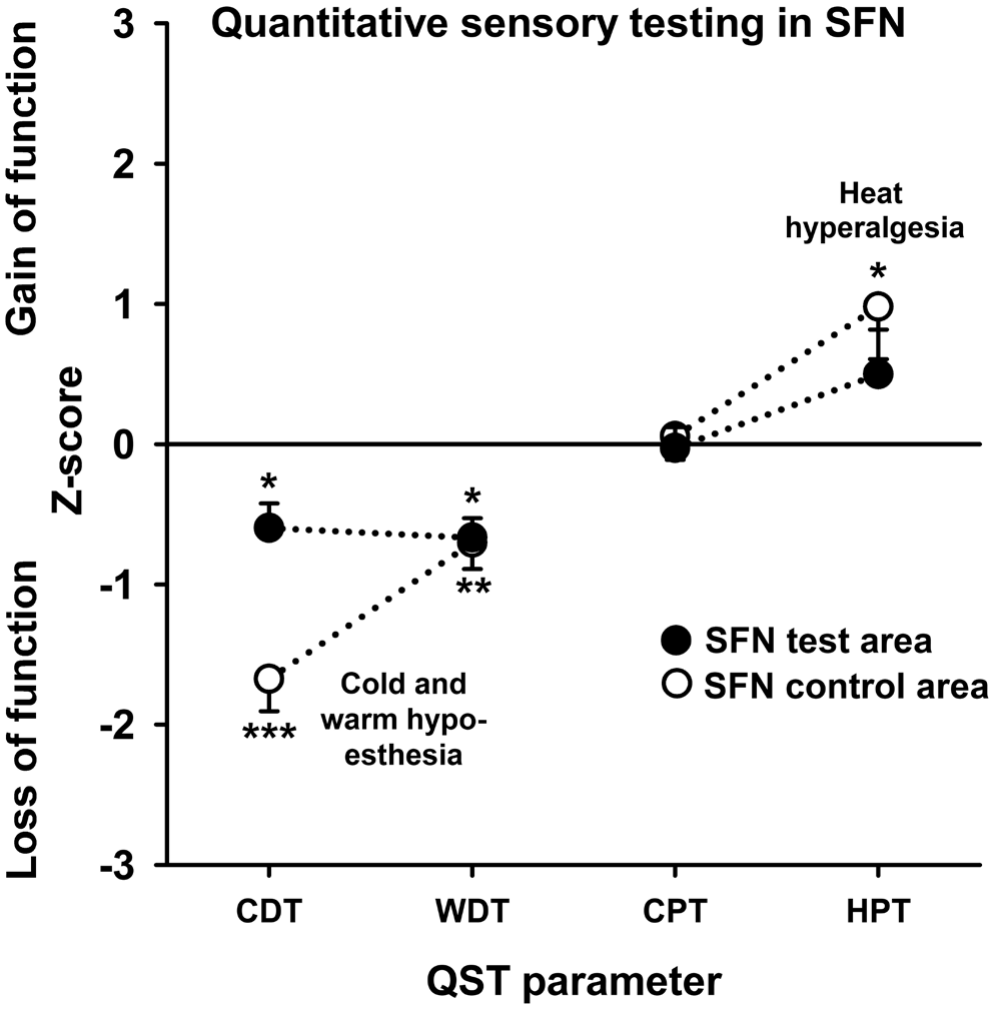
The z‐score QST profiles of SFN patients shows for the most painful test site a thermal detection deficit in line with small fibre dysfunction. This finding is present in a distant non‐ or less painful control area indicating a generalization of SFN. Only for the control region, heat hyperalgesia is present that may be consistent with a peripheral sensitization of the nociceptive system. Asterisks denote levels of significance: *p < 0.05, **p < 0.01***, *p* < 0.001.

QST profiles revealed a significant cold hypoesthesia (CDT test and control), warm hypoesthesia (WDT test and control), and heat hyperalgesia (HPT control) in SFN patients, indicating combined loss and gain of function specifically of C and A‐delta nerve fibres. A potential bias for the interpretation of these results was that a pathological QST profile was part of the inclusion criteria for SFN patients (unlike controls). We conducted t‐tests to assess differences in CDT, WDT, CPT, and HPT between patients with SFN and control subjects matched for age, sex, and body area. (Table 1). Figure 4 illustrates sensory profiles represented by z‐scores.

### 3.4 Explorative subgroup analyses

#### 3.4.1 GLM

For explorative subgroup analyses, we calculated the contrast Hot > Warm for SFN patients with Nav variants and without variants, separately. After that, we compared both groups with each other and with HC, respectively. Note, that none of the analyses survived FDR‐correction for multiple comparisons (*p* < 0.05). Uncorrected explorative GLM analyses can be found in the supplement (Table S5–S9).

#### 3.4.2 Seed to voxel connectivity

For the group comparison of HC subjects and Nav variant carriers (HC > SFN patients with Nav variants), we found significantly higher connections between SMA and right middle frontal gyrus (MFG) for HC compared to patients with Nav variants. Positive t‐values point to an increased SMA‐MFG connection during pain processing for HC. Further, we analysed group differences comparing HC subjects and patients without genetic variants (HC > SFN patients without Nav variants). The analysis revealed significant SMA connectivity to left cerebellum (Crus 2) that was more pronounced in the patients. We further analysed SMA connectivity for SFN patients with‐ and without Nav variants, respectively. The analyses did not reveal any significant findings. Also, for the comparison of Nav variant carriers and patients without variants, no significant group differences were found.

Next, we used the left CN as seed region. The results showed significantly higher connections between left CN and right frontal pole in HC subjects compared to Nav variant carriers (HC > SFN patients with Nav variants). Comparing patients HC subjects and patients without Nav variants (HC > SFN patients without Nav variants) we did not identify significant findings. We did not find significant CN connections for HC subjects and SFN patients, respectively. Also, group comparison did not reveal significant findings. Similarly, neither the analyses for SFN patients with and without Nav variants, respectively, nor the comparison between the subgroups yielded significant results.

We further conducted connectivity analyses with the left insula as seed region. Here, for patients without Nav variants, we found significant insula connectivity with the left SPL. The comparison between these patient subgroups did not reveal a significant finding. Also, the analysis of potential group differences between SFN patients without Nav variants and HC subjects yielded no significant results. Analysing functional connections for HC subjects and SFN patients separately, we did not observe significant findings. Also, the comparison between HC and patients did not reveal significant group differences. For SFN patients with Nav variants, no significant insula connections were identified.

## 4 DISCUSSION

Central pain processing mechanisms may be altered in patients with SFN. To assess these mechanisms, we conducted fMRI during thermal heat pain application in SFN patients and HC. Pain processing brain regions SMA, insula, and CN were defined as seeds for functional connectivity analyses. Analyses revealed a significantly increased connection during pain processing between right SMA and thalamus for SFN patients compared to HC. SMA‐thalamus connectivity correlated significantly with IENFD suggesting a link between peripheral and central pain processing in SFN. Our study provides evidence for altered brain functional connectivity in SFN that may be linked to peripheral mechanisms of the disorder. Further studies are needed to assess the potential role of functional connectivity alterations as a feature for SFN.

In healthy subjects, we identified activation of right SMA; an area that has been associated with pain processing before (Coghill et al., 1999). Traditionally, SMA is thought of as a motor area. However, it is also activated during pain processing studies in which motor function is not explicitly manipulated (Misra & Coombes, 2015; Wager et al., 2013). Further, SMA is reported to be engaged in emotional contexts (Coombes et al., 2012) which is also relevant for pain processing. Therein, SMA may be involved in the muscle coordination that drives force output that is crucial for planning and execution of voluntary movements (Misra & Coombes, 2015) to react to a painful stimulus. We found significantly increased functional connectivity between right SMA and right thalamus in SFN patients compared to HC. As a major component of the pain system, the thalamus is the first relay station receiving and processing nociceptive input (Dostrovsky, 2000). Through the spinothalamic tract (STT), nociceptive inputs are transmitted from the spinal cord to the thalamus (Yen & Lu, 2013). Increased thalamic connectivity may thus reflect an activation of ascending or descending pathways. The lateral and medial pain pathways are the main ascending pathways consisting of a sensory painfulness component (lateral) and a suffering component (medial) comprising autonomic, emotional, and cognitive features, whereas the descending inhibitory pathway is involved in pain suppression (De Ridder et al., 2021). With our approach, we cannot precisely determine which thalamic nuclei were activated. However, peak MNI coordinates [6, −20, 12] of the identified cluster rather indicate an activation of the medial part of the thalamus. Increased SMA‐thalamus coupling may reflect the suffering component of the medial pathway in SFN patients, which can be expressed as anger, depression, or anxiety. Because the medial pathway overlaps with salience and stress networks, it may explain that relevance or meaning can determine the suffering associated with painfulness (De Ridder et al., 2021). Additionally to medial pathway activation, it is possible that the descending pathway (rather anterior thalamic nuclei) is involved as well. The descending system is often deficient in chronic pain disorders (such as fibromyalgia). Since this pathway reflects the capacity of the brain to suppress acute or ongoing pain, a dysfunction of the system normally results in constant pain. In SFN patients, increased SMA‐thalamus connectivity may reflect an activation of this system to compensate for dysfunctional processing.

Altered functional connectivity has been reported in studies focusing on aberrant connectivity as a crucial factor for chronic pain (Cifre et al., 2012; Hemington et al., 2016). Previous research showed enhanced medial thalamus activity after applying innocuous thermal stimuli to patients with heat allodynia, suggesting an involvement of the medial pathway in neuropathic pain (Ab Aziz & Ahmad, 2006; Baron et al., 1999; Casey et al., 2003; Witting et al., 2001). Medial pathway C‐fibres, typically activated by noxious thermal, mechanical, or chemical stimulation, project nociceptive information from the spinal cord (lateral STT) to the thalamus (De Ridder et al., 2021; Yen & Lu, 2013).

Reduced density of peripheral myelinated Aδ‐fibres and unmyelinated C‐fibres is a clinical marker for SFN diagnosis. Interestingly, we found a positive correlation between SMA‐thalamus connectivity and IENFD, indicating a link between reduced fibre density and reduced functional connectivity in SFN patients. A previous diffusion tensor imaging (DTI) study found reduced thalamic white matter connectivity with the insular cortex and sensorimotor areas in SFN patients compared to healthy controls. The degree of fibre degeneration in those SFN patients, measured by IENFD, was linked to the reduction of thalamic connectivity (Chao et al., 2021). Although they assessed structural connectivity, our results seem to contradict the findings from that previous study. We assume that increased connectivity between SMA and thalamus reflects a functional over‐compensation during painful stimulation, which is not necessarily linked to potential brain structural deficits in SFN patients. In our study, we found reduced IENFD in SFN patients. Thereby, higher IENFD was associated with higher thalamic connectivity. This may point to a link between medial pathway activation and the amount of C‐fibres and Aδ‐fibres involved in processing of noxious stimuli. One hypothesis might be that the suffering component and the related medial pathway activation is more pronounced in SFN patients with a lower amount of loss of small nerve fibre function. Moreover, centrally mediated hypoesthesia rather than actual small nerve fibre damage has been described before, where cortical shrinkage in ongoing pain was discussed as a possible source of diminished sensory processing. Not the number but the altered input from skin nociceptive afferents might be the important trigger to cortical nociplastic connectivity. This is in line with experiments in rodents where cortical activation following input from capsaicin‐sensitive C‐fibres has been shown to induce inhibitory control over the excitability of non‐nociceptive somatosensory cortical neurons (Calford & Tweedale, 1991). We assume less disturbed C‐fibre input when the numbers and function of skin C‐fibres is better preserved. Mechanisms such as secondary suppression and reorganization (shrinkage) of the somatosensory cortex, as described in complex regional pain syndrome (Pleger et al., 2005), may also influence the observed brain connectivity in the present study. A previous fMRI study on healthy subjects found that heat pain activated pain‐related brain regions and increased connectivity with the somatosensory cortex. The connectivity strength was positively correlated with IENFD suggesting interactions between cerebral pain circuits and peripheral nociceptive fibres (Tseng et al., 2015). Another fMRI study on SFN patients examined central changes after peripheral nerve degeneration, finding decreased connectivity of the anterior cingulate cortex (ACC) with pain processing regions during noxious heat stimulation, which correlated with the degree of nerve fibre reduction (Hsieh et al., 2015). These studies suggest a link between peripheral nerve fibres and brain functional changes in SFN patients.

It would be beneficial for future studies to consider which specific nerve fibres are involved. In neuropathy, mechano‐insensitive fibres (CMi) get sensitized and may become hyperexcitable (Ackerley & Watkins, 2018). Mechano‐responsive fibres (CM) are known to be relevant for an individuals' heat pain detection (Tillman et al., 1995), while CMi fibres require higher thresholds (such as approx. 47–48°C) (Schmelz et al., 2000). In our patient cohort, however, the mean temperature applied during fMRI was 44°C. Moreover, CM fibres are more responsible for stimuli coming from the outside of the body causing a motor reaction to adapt (Ørstavik et al., 2006). Therefore, in our study, an involvement of CM fibres during the application of experimental pain is more likely. Only CM fibres are reflected by IENFD, but not CMi fibres (Ørstavik et al., 2006), suggesting there may be no direct link between SMA‐thalamus connectivity and the amount of IENFD. However, another hypothesis might be that in SFN patients CMi fibres, a key player in neuropathic pain (Forstenpointner et al., 2019), are more likely to activate the medial pathway due to chronic pain states. Since these interpretations remain speculative, they need further validation by studies combining both, methods measuring brain activity (e.g., fMRI) and approaches assessing the conduction of nerve signals (action potentials) in peripheral nerve fibres such as microneurography (e.g., Namer et al., 2009). It is important to note that, due to the fact that the correlational results did not survive correction for multiple testing, they must be interpreted with caution.

Explorative connectivity analyses were performed in a subsample of SFN patients with rare heterozygous missense Nav variants. We found significant differences between patients with Nav variants compared to HC. Also, we identified connectivity differences between patients without Nav variants and HC. Comparing SFN patients with and without Nav variants, we found increased activity in left SFG and PCL for Nav variant carriers. SFG is associated with pain anticipation (Palermo et al., 2015), pain catastrophizing (Gracely et al., 2004) and the affective experience of pain (Fulbright et al., 2001). PCL has been linked to increased sensitivity to thermal stimuli (Erpelding et al., 2012) and to the emotional modulation of pain (Roy et al., 2009). Taken together, patients with Nav variants were more likely to activate brain regions involved in the emotional component of pain processing compared to patients without Nav variants. However, caution should be exercised when interpreting these findings, as our exploratory subgroup analyses were conducted with just nine patients carrying Nav variants. We acknowledge that this subset is notably small, and thus, these analyses should be regarded as preliminary. Furthermore, it is crucial to distinguish between disease‐causing variants, disease‐contributing variants, and variants of uncertain significance (Le Cann et al., 2021; Waxman et al., 2014). Some studies suggest that specific, infrequent variants in SFN might function as risk factors that contribute to, rather than directly cause, the disease (Sopacua et al., 2019). The precise pathogenic significance of these variants remains uncertain and requires further investigation.

### 4.1 Limitations

Concerning WDT and HPT measured immediately before the fMRI session, we did not find significant differences between HC and SFN patients. One explanation might be that we applied heat stimulation to the same body part for all participants. Since patients' symptoms may occur in different body parts, pain‐related brain activity may also vary between stimulation applied to affected or unaffected body regions. In our sample, six patients reported feelings of insensitivity or hypersensitivity in the test area. However, the analyses did not reveal significant group differences when we excluded these patients. Follow‐up studies may consider analysing differences between pain‐related brain activity following stimulation in affected and unaffected body regions, respectively. Results from the GLM analyses did not survive correction for multiple comparisons (FDR, *p* < 0.05). Thus, these findings need to be interpreted cautiously. Regarding correlation analyses, it is important to highlight that multiple tests were conducted, but none of the correlations remained significant after adjusting for multiple comparisons. The statistical power of the analyses on patients with rare Nav variants is limited due to the small subsample of just nine patients. These variants are not confirmed as pathogenic; they are considered variants of uncertain significance. Before drawing definitive conclusions from these initial findings, it is crucial to validate the results in larger sample sizes.

## 5 CONCLUSION

Our study sheds light on potential alterations in central pain processing mechanisms of experimentally applied thermal stimuli among patients with SFN. Using fMRI and thermal heat pain application, we identified increased connectivity between specific brain regions, notably right SMA and thalamus (contralateral to heat stimulation), in SFN patients compared with healthy subjects. This increased connectivity correlated with IENFD, suggesting a potential connection between peripheral and central processing in SFN. Moreover, when exploring specific genetic variants in SFN patients, we observed distinct connectivity patterns. However, given the unclear significance of the variants and the small sample size, the potential effect of genetic variants in Nav channels on functional connectivity requires further validation. In conclusion, functional connectivity changes may be associated with SFN, emphasizing that these changes may result from peripheral alterations causing aberrant central processing. This understanding may be crucial for assessing their impact on painful symptoms and therapy response. Further research is necessary to confirm and expand upon these results.

## AUTHOR CONTRIBUTIONS

This study was designed by U.H. and H.J. The experiments were performed by S.S. and T.K. The data was analysed by S.S. and T.K., and the results were critically examined by all authors. S.S. had a primary role in preparing the manuscript, which was edited by all authors. All authors have approved the final version of the manuscript and agree to be accountable for all aspects of the work.

## FUNDING INFORMATION

This work was funded by the Deutsche Forschungsgemeinschaft (DFG, German Research Foundation)—368,482,240/GRK2416 (‘RTG 2416 MultiSenses – MultiScales’), by a grant from the Interdisciplinary Centre for Clinical Research within the faculty of Medicine at the RWTH Aachen University (IZKF TN1‐8/IA 532008, IZKF TN1‐6/IA 532006, IZKF TN1‐1/IA 532001, IZKF TN1‐2/IA 532002, IZKF TN1‐9/IA 532009) also to BNs research group and the Brain Imaging Facility of the Interdisciplinary Centre for Clinical Research (IZKF) within the faculty of Medicine at the RWTH Aachen University, Germany. This work was supported under the framework of international cooperation program managed by the National Research Foundation of Korea (2023K2A9A2A22000113).

## CONFLICT OF INTEREST STATEMENT

The authors declare no conflicts of interest related to the present study.

## PLAGIARISM DECLARATION

We hereby declare that this paper is our own work, except where acknowledged, and has not been submitted elsewhere.

## Supporting information


Table S1.

Table S2.

Table S3.

Table S4.

Table S5.

Table S6.

Table S7.

Table S8.

Table S9.

## Data Availability

The data from this study is available on request from the corresponding author. The data is not publicly available due to it containing information that could compromise the participant's privacy.
