# Supplementary material for: Painful stimulation increases functional connectivity between supplementary motor area and thalamus in patients with small fibre neuropathy
Source: Eur J Pain. 2024 Aug 28;29(2):e4720. doi: 10.1002/ejp.4720 (PMC11671338; doi:10.1002/ejp.4720)
Supplement: Supplementary file 1 — Table S1. [file EJP-29-0-s009.docx]

| **Patient** | **Medication** | **Doses** |
| --- | --- | --- |
| #1 | NA | NA |
| #2 | Pregabalin | 75mg |
| #3 | NA | NA |
| #4 | NA | NA |
| #5 | NA | NA |
| #6 | Amitryptilin | 50mg |
| #7 | Pregabalin | 25mg |
| #8 | Amitryptilin | 7mg |
| #9 | Duloxetin | 60mg |
| #10 | Pregabalin | 100-150mg |
| #11 | Pregabalin | 100mg |
| #12 | NA | NA |
| #13 | Novalgin | NA |
| #14 | Carbamazepin | 200mg |
| #15 | Gabapentin | NA |
| #16 | NA | NA |
| #17 | NA | NA |
| #18 | Novalgin | NA |
| #19 | NA | NA |
| #20 | NA | NA |
| #21 | NA | NA |
| #22 | NA | NA |
| #23 | NA | NA |
| #24 | NA | NA |
| #25 | Gabapentin | 800mg |
| #26 | Carbamazepin | 400mg |
| #27 | Duloxetin | 30mg |
| #28 | NA | NA |
| #29 | Pregabalin | 75mg |
| #30 | NA | NA |
| #31 | NA | NA |
| #32 | NA | NA |
| **Abbreviations.** NA, not available; mg, milligram | | |

**Table S1**. Medication in the patient sample.
