# Supplementary material for: Painful stimulation increases functional connectivity between supplementary motor area and thalamus in patients with small fibre neuropathy
Source: Eur J Pain. 2024 Aug 28;29(2):e4720. doi: 10.1002/ejp.4720 (PMC11671338; doi:10.1002/ejp.4720)
Supplement: Supplementary file 2 — Table S2. [file EJP-29-0-s005.docx]

| **Patient** | **Channel** | **Gene** | **gDNA (GRCh38)** | **Transcript** | **cDNA** | **Protein** |
| --- | --- | --- | --- | --- | --- | --- |
| #1 | Nav1.9 | *SCN11A* | Chr3:g.38847191A>G | NM_001349253.2 | c.4879T>C | p.(Tyr1627His) |
| #2 | Nav1.9 | *SCN11A* | Chr3:g.38950166T>G | NM_001349253.2 | c.197A>C | p.(Tyr66Ser) |
| #3 | Nav1.9 | *SCN11A* | Chr3:g.38950166T>G | NM_001349253.2 | c.197A>C | p.(Tyr66Ser) |
| #4 | Nav1.8 | *SCN10A* | Chr3:g.38714058G>T | NM_006514.4 | c.3704C>A | p.(Ala1235Glu) |
|  | Nav1.7 | *SCN9A* | Chr2:g.166226660A>C | NM_002977.3 | c.4272T>G | p.(Ile1424Met) |
| #5 | Nav1.8 | *SCN10A* | Chr3:g.38793970C>A | NM_006514.4 | c.41G>T | p.(Arg14Leu) |
| #6 | Nav1.8 | *SCN10A* | Chr3:g.38793970C>A | NM_006514.4 | c.41G>T | p.(Arg14Leu) |
| #7 | Nav1.7 | *SCN9A* | Chr2:g.166272748T>C | NM_002977.3 | c.2969A>G | p.(Tyr990Cys) |
| #8 | Nav1.9 | *SCN11A* | Chr3:g.38904083G>T | NM_001349253.2 | c.1624C>A | p.(Pro542Thr) |
| #9 | Nav1.7 | *SCN9A* | Chr2:g.166280452T>C | NM_002977.3 | c.2215A>G | p.(Ile739Val) |

**Table S2**. Genetic variants in Nav channels in the patient sample.
