# Supplementary material for: Painful stimulation increases functional connectivity between supplementary motor area and thalamus in patients with small fibre neuropathy
Source: Eur J Pain. 2024 Aug 28;29(2):e4720. doi: 10.1002/ejp.4720 (PMC11671338; doi:10.1002/ejp.4720)
Supplement: Supplementary file 3 — Table S3. [file EJP-29-0-s003.docx]

**Table S3**. Significant clusters for the main effect of temperature.

| Region | k | Peak MNI coordinates | | | Peak T-value^*^ |
| --- | --- | --- | --- | --- | --- |
|  |  | x | y | z |  |
| *HC: Hot > Warm* | | | | | |
| R SMA | 1820 | 4 | -8 | 64 | 4.50 |
| L insula | 77 | -38 | -6 | 2 | 4.21 |
| R insula | 166 | 34 | 4 | 10 | 4.08 |
| L SMG | 82 | -58 | -20 | 22 | 3.75 |
| L cerebellum | 52 | -20 | -58 | -18 | 3.73 |
| R RO | 52 | 54 | 4 | 16 | 3.67 |
| R SMG | 157 | 54 | -32 | 44 | 3.59 |
| L CN | 40 | -14 | 4 | 10 | 3.55 |
| L cerebellum | 39 | -2 | -56 | -6 | 3.53 |
| *SFN patients: Hot > Warm* | | | | | |
| L insula | 748 | -56 | -20 | 24 | 4.87 |
| L SFG | 413 | -24 | -6 | 62 | 4.59 |
| R RO | 206 | 54 | -20 | 24 | 4.40 |
| R RO | 242 | 50 | 6 | 12 | 4.19 |
| R insula | 21 | 32 | 24 | 4 | 3.56 |
| **Abbreviations.**  R, right; L, left; HC, healthy controls; SFN, small-fiber neuropathy patients; SMA, supplementary motor area; SMG, supramarginal gyrus; RO, Rolandic operculum; CN, caudate nucleus; SFG, superior frontal gyrus  **Notes.** ^*^Height threshold T = 3.158 (*p* < 0.001, uncorrected); Extent threshold k = 20 voxels | | | | | |
