# Supplementary material for: Painful stimulation increases functional connectivity between supplementary motor area and thalamus in patients with small fibre neuropathy
Source: Eur J Pain. 2024 Aug 28;29(2):e4720. doi: 10.1002/ejp.4720 (PMC11671338; doi:10.1002/ejp.4720)
Supplement: Supplementary file 4 — Table S4. [file EJP-29-0-s008.docx]

**Table S4.** Correlations between SMA-thalamus connectivity and clinical parameters.

| Clinical parameters | Uncorrected results | | Bonferroni corrected results |
| --- | --- | --- | --- |
|  | *r* | *p* | *p* |
| Symptom duration | .004 | 0.982 | > .999 |
| IENFD | .399 | 0.044* | 0.616 |
| NRS | -.108 | 0.585 | > .999 |
| painDETECT | -.071 | 0.706 | > .999 |
| CDT test | -.098 | 0.955 | > .999 |
| CDT control | .177 | 0.620 | > .999 |
| WDT test | -.089 | 0.611 | > .999 |
| WDT control | .146 | 0.359 | > .999 |
| CPT test | .120 | 0.648 | > .999 |
| CPT control | .206 | 0.451 | > .999 |
| HPT test | -.177 | 0.536 | > .999 |
| HPT control | .243 | 0.285 | > .999 |
| **Abbreviations.** IENFD, intraepidermal nerve fiber density; NRS, numeric ratings scale (pain); CDT, cold detection threshold; WDT, warm detection threshold; CPT, cold pain threshold; HPT, heat pain threshold  **Notes.** * *p* < .05 | | | |
