# Supplementary material for: Painful stimulation increases functional connectivity between supplementary motor area and thalamus in patients with small fibre neuropathy
Source: Eur J Pain. 2024 Aug 28;29(2):e4720. doi: 10.1002/ejp.4720 (PMC11671338; doi:10.1002/ejp.4720)
Supplement: Supplementary file 5 — Table S5. [file EJP-29-0-s006.docx]

**Table S5**. SFN patients with Nav-variants: Significant clusters for the main effect of temperature (Hot > Warm).

| Region | k | Peak MNI coordinates | | | Peak T-value^*^ |
| --- | --- | --- | --- | --- | --- |
|  |  | x | y | z |  |
| L SFG | 503 | -30 | -4 | 62 | 5.04 |
| L SMG | 61 | -46 | -30 | 32 | 4.46 |
| R SPL | 49 | 20 | -52 | 68 | 3.62 |
| R SPL | 25 | 40 | -46 | 62 | 3.59 |
| **Abbreviations.**  R, right; L, left; SFG, superior frontal gyrus; SMG, supramarginal gyrus; SPL, superior parietal lobule  **Notes.** ^*^Height threshold T = 3.232 (*p* < 0.001, uncorrected); Extent threshold k = 20 voxels | | | | | |
