# Supplementary material for: Painful stimulation increases functional connectivity between supplementary motor area and thalamus in patients with small fibre neuropathy
Source: Eur J Pain. 2024 Aug 28;29(2):e4720. doi: 10.1002/ejp.4720 (PMC11671338; doi:10.1002/ejp.4720)
Supplement: Supplementary file 6 — Table S6. [file EJP-29-0-s004.docx]

**Table S6**. SFN patients without Nav-variants: Significant clusters for the main effect of temperature (Hot > Warm).

| Region | k | Peak MNI coordinates | | | Peak T-value^*^ |
| --- | --- | --- | --- | --- | --- |
|  |  | x | y | z |  |
| L RO | 46 | -52 | -2 | 10 | 3.85 |
| L insula | 36 | -32 | 16 | 4 | 3.85 |
| L PostCG | 22 | -56 | -18 | 24 | 3.64 |
| R RO | 23 | 54 | -16 | 22 | 3.59 |
| **Abbreviations.**  R, right; L, left; RO, Rolandic operculum; PostCG, postcentral gyrus  **Notes.** ^*^Height threshold T = 3.170 (*p* < 0.001, uncorrected); Extent threshold k = 20 voxels | | | | | |
