# Supplementary material for: Painful stimulation increases functional connectivity between supplementary motor area and thalamus in patients with small fibre neuropathy
Source: Eur J Pain. 2024 Aug 28;29(2):e4720. doi: 10.1002/ejp.4720 (PMC11671338; doi:10.1002/ejp.4720)
Supplement: Supplementary file 7 — Table S7. [file EJP-29-0-s001.docx]

**Table S7**. Healthy controls vs. SFN patients with Nav-variants: Significant clusters for the main effect of temperature (Hot > Warm).

| Region | k | Peak MNI coordinates | | | Peak T-value^*^ |
| --- | --- | --- | --- | --- | --- |
|  |  | x | y | z |  |
| *Healthy controls > SFN patients with Nav-variants* | | | | | |
| No voxel survived | | | | | |
| *SFN patients with Nav-variants > Healthy controls* | | | | | |
| L MTG | 28 | -44 | -30 | -2 | 3.51 |
| **Abbreviations.**  R, right; L, left; MTG, middle temporal gyrus  **Notes.** ^*^Height threshold T = 3.170 (*p* < 0.001, uncorrected); Extent threshold k = 20 voxels | | | | | |
