# Supplementary material for: Painful stimulation increases functional connectivity between supplementary motor area and thalamus in patients with small fibre neuropathy
Source: Eur J Pain. 2024 Aug 28;29(2):e4720. doi: 10.1002/ejp.4720 (PMC11671338; doi:10.1002/ejp.4720)
Supplement: Supplementary file 8 — Table S8. [file EJP-29-0-s007.docx]

**Table S8**. Healthy controls vs. SFN patients without Nav-variants: Significant clusters for the main effect of temperature (Hot > Warm).

| Region | | k | Peak MNI coordinates | | | | Peak T-value^*^ |
| --- | --- | --- | --- | --- | --- | --- | --- |
|  |  |  | x | y | z | |  |
| *Healthy controls > SFN patients without Nav-variants* | | | | | | | |
| L MCC | 46 | | -2 | -6 | | 44 | 3.75 |
| *SFN patients without Nav-variants > Healthy controls* | | | | | | | |
| No voxel survived | | | | | | | |
| **Abbreviations.**  R, right; L, left; MCC, middle cingulate cortex  **Notes.** ^*^Height threshold T = 3.170 (*p* < 0.001, uncorrected); Extent threshold k = 20 voxels | | | | | | | |
