# Supplementary material for: Painful stimulation increases functional connectivity between supplementary motor area and thalamus in patients with small fibre neuropathy
Source: Eur J Pain. 2024 Aug 28;29(2):e4720. doi: 10.1002/ejp.4720 (PMC11671338; doi:10.1002/ejp.4720)
Supplement: Supplementary file 9 — Table S9. [file EJP-29-0-s002.docx]

**Table S9**. SFN patients with Nav-variants vs. SFN patients without Nav-variants: Significant clusters for the main effect of temperature (Hot > Warm).

| Region | | k | Peak MNI coordinates | | | | Peak T-value^*^ |
| --- | --- | --- | --- | --- | --- | --- | --- |
|  |  |  | x | y | z | |  |
| *SFN patients with Nav-variants > SFN patients without Nav-variants* | | | | | | | |
| L SFG | 62 | | -16 | 66 | | 22 | 4.34 |
| L PCL | 92 | | -4 | -26 | | 70 | 3.71 |
| *SFN patients without Nav-variants > SFN patients with Nav-variants* | | | | | | | |
| No voxel survived | | | | | | | |
| **Abbreviations.**  R, right; L, left; SFG, superior frontal gyrus; PCL, paracentral lobule  **Notes.** ^*^Height threshold T = 3.232 (*p* < 0.001, uncorrected); Extent threshold k = 20 voxels | | | | | | | |
